# Exploring Healthy Aging Challenges Among Veterans and Non-Veterans in Permanent Supportive Housing

**DOI:** 10.1093/geroni/igaf122.4115

**Published:** 2025-12-31

**Authors:** Jun Heo, Janelle Cardenas, Teena Ellison, April Aiken, Anita Souza

**Affiliations:** University of Washington School of Nursing, Kent, Washington, United States; Compass Housing Alliance, Seattle, Washington, United States; Compass Housing Alliance, Seattle, Washington, United States; Compass Housing Alliance, Seattle, Washington, United States; University of Washington, Seattle, Washington, United States

## Abstract

As individuals experiencing homelessness age, their healthcare needs become increasingly complex. Adults over 50 represent the fastest-growing group among the homeless population, yet the population is highly heterogeneous, and the factors influencing their ability to age well are underexplored. This study examined barriers to healthcare and housing stability among older adults with lived experience of homelessness, focusing on differences between veterans and non-veterans. We used a qualitative design and developed a semi-structured interview guide in collaboration with Compass Housing Alliance. We recruited 20 participants over age 50 who had recently exited homelessness and were residing in Permanent Supportive Housing (PSH). We conducted 45-minute interviews with veterans (n = 10) and non-veterans (n = 10), asking about daily life, healthcare access, social connection, use of support services, and perceptions of aging well. Participants also completed a brief demographic survey. Interviews were recorded, transcribed, and coded to identify key themes. While both groups reported similar overall needs and behaviors, one notable barrier across participants was untreated dental issues, which significantly impacted well-being. Veterans more frequently described challenges with social adjustment and reintegration, whereas non-veterans more often reported difficulty accessing consistent healthcare. These subgroup-specific differences reflect unique structural vulnerabilities shaped by service systems and lived histories. By drawing on lived experience, this study highlights important distinctions within the aging homeless population. Findings can inform policies and services that support healthy aging in Permanent Supportive Housing, helping healthcare providers and housing organizations tailor interventions to the needs of diverse older adults who have experienced homelessness.

